# Genomic epidemiology of *Salmonella Typhimurium* and its monophasic variants in Southern China: A spatiotemporal and source attribution analysis

**DOI:** 10.1016/j.onehlt.2025.101299

**Published:** 2025-12-15

**Authors:** Ningbo Liao, Shunxiong Lei, Chengwei Liu, Shengnan Tang, Silu Peng

**Affiliations:** a School of Food Science & Engineering, Jiangxi Agricultural University, Nanchang 330045, PR China; b Jiangxi Provincial Key Laboratory of Major Epidemics Prevention and Control, Key Laboratory of Nutrition Diet and Health of Jiangxi Provincial Health Commission, Jiangxi Provincial Center for Disease Control and Prevention, 555 East Beijing Road, Nanchang 330029, China; c The Department of Gastroenterology, First Affiliated Hospital of Nanchang University, Nanchang, Jiangxi 330006, PR China

**Keywords:** *Salmonella Typhimurium*, Genomic epidemiology, Foodborne disease, Antimicrobial resistance, Zoonotic transmission, Whole-genome sequencing

## Abstract

*Salmonella Typhimurium* and its monophasic variants are major contributors to foodborne illnesses globally, with zoonotic transmission posing significant public health risks. In southern China, persistent salmonellosis cases linked to poultry and pork highlight the need for advanced genomic tools to trace contamination sources and understand transmission dynamics. This study integrates whole-genome sequencing (WGS) and spatiotemporal data to investigate the molecular epidemiology of Salmonella in Jiangxi Province, a region with high incidence of foodborne salmonellosis. Analysis of 206 Salmonella isolates (2015–2021) revealed dominant sequence types (ST34, ST19, ST155, and ST469) associated with human clinical cases and food sources. High-resolution single nucleotide polymorphism (SNP) phylogenetic analysis revealed well-supported, monophasic clades corresponding to the major sequence types. This analysis provided strong genomic evidence for zoonotic transmission, with human clinical isolates being genetically almost identical (≤5 SNPs) to isolates from poultry (ST34/ST19) and pork (ST155/ST469) sources. Clonal clusters of monophasic Typhimurium variants (77.9 % of ST34 isolates) exhibited widespread geographic distribution across 11 prefectures, and the high genetic similarity among isolates suggests potential cross-regional transmission through contaminated food supply chains. High antimicrobial resistance (AMR) rates were detected against ampicillin (68.0 %), tetracycline (61.0 %), and sulfamethoxazole-trimethoprim (54.0 %), with multidrug-resistant (MDR) strains (60.2 %) prevalent in clinical and food-derived isolates. ST34 exhibited the highest MDR prevalence (75.4 %), driven by the presence of Salmonella Genomic Island 1 (SGI1) in many isolates. The β-lactamase gene blaTEM-1 was most prevalent (60.7 %), followed by tet(A) (54.4 %), and sul2 (47.6 %). Point mutations in the quinolone resistance-determining region (QRDR), specifically in gyrA, were identified as the primary mechanism for ciprofloxacin resistance. Spatial clustering identified significant clusters in live poultry markets, slaughterhouses, and retail meat, emphasizing the role of foodborne zoonotic reservoirs. Findings advocate for strengthened One Health interventions, including enhanced AMR monitoring, targeted food safety regulations, and real-time WGS-based surveillance to mitigate zoonotic transmission risks in southern China.

## 1 Introduction

*Salmonella enterica* serovar Typhimurium and its monophasic variants (1,4, [5],12:i:-) rank among the most pervasive foodborne pathogens worldwide, contributing substantially to the global burden of gastroenteritis, invasive bloodstream infections, and antimicrobial resistance (AMR)-related fatalities [1]. These pathogens thrive in diverse reservoirs, with zoonotic transmission via contaminated poultry, pork, and livestock products driving recurrent outbreaks in both industrialized and resource-limited settings [2]. The emergence of monophasic Typhimurium strains—phenotypically lacking phase 2 flagellar antigens but retaining the virulence repertoire of biphasic ancestors—has complicated traditional surveillance, as these variants exploit gaps in serotyping-based detection systems while demonstrating enhanced adaptability to agricultural niches [3].

Southern China, a hub of intensive livestock production and high-density live animal markets, faces persistent *Salmonella* transmission risks, with Jiangxi Province reporting elevated rates of human salmonellosis linked to poultry and pork consumption [4,5]. Despite epidemiological evidence implicating food supply chains in outbreak propagation, critical knowledge gaps persist regarding strain-specific transmission routes, the genomic basis of AMR escalation, and the spatial dynamics of cross-regional pathogen dissemination. Conventional surveillance methods, reliant on serotyping and pulsed-field gel electrophoresis (PFGE), lack the resolution to disentangle complex clonal relationships or identify emerging variants with public health significance.

Whole-genome sequencing (WGS) has revolutionized molecular epidemiology by enabling high-resolution phylogenetic reconstruction, precise source attribution, and real-time tracking of AMR gene dissemination [6]. However, its application remains underutilized in China's food safety ecosystems, particularly in regions where fragmented surveillance systems and unregulated antibiotic use in agriculture exacerbate AMR risks [7]. In Jiangxi, a province characterized by interlinked poultry farms, slaughterhouses, and urban retail networks, the convergence of anthropogenic and environmental factors creates ideal conditions for *Salmonella* persistence and evolution [8,9].

This study bridges critical gaps by integrating WGS-based genomic surveillance, including high-resolution SNP analysis, spatiotemporal clustering, and phenotypic AMR profiling to dissect the epidemiology of *Salmonella Typhimurium* variants across Jiangxi Province from 2015 to 2021. We characterize dominant sequence types (STs) circulating at the human-livestock interface, investigate potential transmission links, and map the genomic landscape of resistance determinants across clinical, poultry, and pork isolates. Our findings expose the pervasive dissemination of multidrug-resistant (MDR) clones through commercial food networks, underscoring the urgency of One Health interventions to disrupt zoonotic transmission cycles. By correlating genomic data with geospatial hotspots, this work establishes a framework for preemptive surveillance and informs evidence-based policies to mitigate AMR spread in southern China's food production systems.

## 2 Materials and methods

### 2.1 Sample collection and bacterial isolation

A total of 206 *Salmonella enterica* isolates were included in this study, prospectively collected from Jiangxi Province, China, between 2015 and 2021. These isolates were recovered from poultry (*n* = 116, 56.3 %; cloacal swabs and retail poultry meat), pork (*n* = 59, 28.6 %; carcass swabs and retail pork products), and human clinical cases (*n* = 31, 15.1 %; stool samples from diarrheal patients). A detailed breakdown of isolates by source and year is provided in Supplementary Table S1. Samples were geographically distributed across 11 prefectures, with spatial coordinates recorded using GPS-enabled devices. Bacterial isolation followed ISO 6579:2017 protocols: samples were enriched in buffered peptone water (24 h, 37 °C), selectively cultured on Xylose Lysine Deoxycholate (XLD) and *Salmonella-Shigella* (SS) agar, and confirmed via biochemical profiling (triple sugar iron agar, lysine decarboxylase, and urease tests) [10]. Species confirmation was performed using *invA*-specific PCR with primers 5′-GTGAAATTATCGCCA CGTTCGGGCAA-3′ (forward) and 5′-TCATCGCACCGTCAAAGGAACC-3′ (reverse) [11].

### 2.2 Whole-genome sequencing and bioinformatic analysis

Genomic DNA was extracted using the QIAamp DNA Mini Kit (Qiagen) and quantified via Qubit 4.0 Fluorometer [12]. Libraries were prepared with the Nextera XT DNA Library Prep Kit and sequenced on the Illumina NovaSeq 6000 platform (150-bp paired-end reads; target coverage ≥50×) [13]. Raw sequencing reads from a representative subset of 54 isolates, selected to encompass the full diversity of sequence types, serovars, isolation sources, and antimicrobial resistance profiles observed in this study, have been deposited in the NCBI Sequence Read Archive (SRA) under BioProject accession PRJNA1370177. A comprehensive list of all 206 isolates, including detailed metadata and identification of the deposited genomes, is provided in Supplementary Table S1. Raw reads were quality-trimmed using Trimmomatic v0.39 and assembled into contigs with SPAdes v3.15.0 (k-mer sizes: 21, 33, 55, 77). Sequence types (STs) were determined via the Achtman 7-gene multilocus sequence typing (MLST) scheme using the Salmonella EnteroBase database [14]. Serotypes were predicted in silico using SeqSero2 v1.2.1 (k-mer-based antigen gene detection) and validated via traditional slide agglutination (Kauffmann-White scheme) [15]. Discordant isolates underwent phase inversion assays to resolve monophasic variants (e.g., fljB deletion screening) [16]. Acquired antimicrobial resistance (AMR) genes were identified using ResFinder v4.1 (90 % identity, 60 % coverage thresholds) and ABRicate v1.0.1, which utilize curated databases to detect known resistance determinants [17]. Genomes were also screened for the presence of Salmonella Genomic Island 1 (SGI1) and its variants using SGI-Finder. Mutations in the quinolone resistance-determining regions (QRDR) of *gyrA, gyrB, parC*, and *parE* were identified by comparing the assembled contigs against the reference genes from *S. typhimurium* LT2 (AE006468.2) and confirmed to be resistance-associated based on established literature for specific amino acid substitutions [18].

### 2.3 Phylogenetic analysis

To infer high-resolution phylogenetic relationships, a core genome single nucleotide polymorphism (SNP) analysis was performed. Raw sequencing reads were first trimmed for quality and adapter content using Trimmomatic v0.39 [19]. The cleaned reads were then mapped to the *S. typhimurium* LT2 reference genome (GenBank accession: AE006468.2) using the BWA-MEM algorithm v0.7.17.

Variant calling was performed using the GATK v4.2 toolkit. SNPs were initially called with HaplotypeCaller and consolidated using GenomicsDBImport and GenotypeGVCFs. To ensure high-confidence variant calls, SNPs were filtered using the following stringent criteria: QUAL >100, Quality by Depth (QD) > 2.0, Mapping Quality (MQ) > 40.0, and read depth (DP) > 10. Regions of the genome associated with mobile genetic elements or predicted to be recombinant using Gubbins v3.2.1 were excluded to mitigate the impact of horizontal gene transfer on the phylogeny. The remaining high-quality, core genome SNPs were concatenated into a single alignment for all isolates.

A maximum-likelihood (ML) phylogenetic tree was constructed from the core SNP alignment using IQ-TREE v2.2.0. The best-fit nucleotide substitution model was first determined automatically by IQ-TREE's ModelFinder component. The ML tree was then inferred under this model, with branch support assessed using 1000 ultrafast bootstrap replicates [19]. The final tree was visualized and annotated with metadata using the interactive Tree Of Life (iTOL) v6 web server.

### 2.4 Antimicrobial susceptibility testing

Susceptibility to 10 antimicrobial agents (AMP, ampicillin; TET, tetracycline; SXT, sulfamethoxazole-trimethoprim; CHL, chloramphenicol; CIP, ciprofloxacin; CTX, cefotaxime; GEN, gentamicin; NAL, nalidixic acid; STR, streptomycin; KAN, kanamycin) was assessed via Kirby-Bauer disk diffusion on Mueller-Hinton agar, following CLSI M100-S31 guidelines [20]. Zone diameters were interpreted using CLSI breakpoints, with *Escherichia coli* ATCC 25922 as the quality control strain. Multidrug resistance (MDR) was defined as resistance to ≥3 antimicrobial classes [21]. Intermediate results were classified as susceptible for binary analysis.

### 2.5 Geospatial and statistical analysis

Spatiotemporal clustering was analyzed using SaTScan v9.6 with a discrete Bernoulli model (maximum spatial cluster size: 50 % population risk; temporal window: 2015–2021) [22]. Geographic distributions were visualized using QGIS v3.22.3, with bubble plots scaled to isolate density [23]. Statistical analyses were performed in R v4.1.0 and Python v3.9. Categorical associations (ST-source correlations, serotype concordance) were assessed via chi-square or Fisher's exact tests, with *p*-values <0.05 considered significant [9]. Temporal trends in AMR rates were evaluated using Cochran-Armitage test for linear trend. A multivariate logistic regression model was constructed to identify factors associated with MDR [24], with results, including odds ratios and 95 % confidence intervals, provided in Supplementary Table S2. Hierarchical clustering (Euclidean distance, Ward's linkage) and heatmaps were generated using Complex Heatmap (R) and seaborn (Python). Spatial autocorrelation was quantified via Moran's I statistic [25]. All visualizations adhered to STRIVE guidelines for epidemiological reporting [26].

## 3 Results

### 3.1 Genetic diversity, phylogenetic structure, and source association of *Salmonella* lineages

**Fig. 1:**
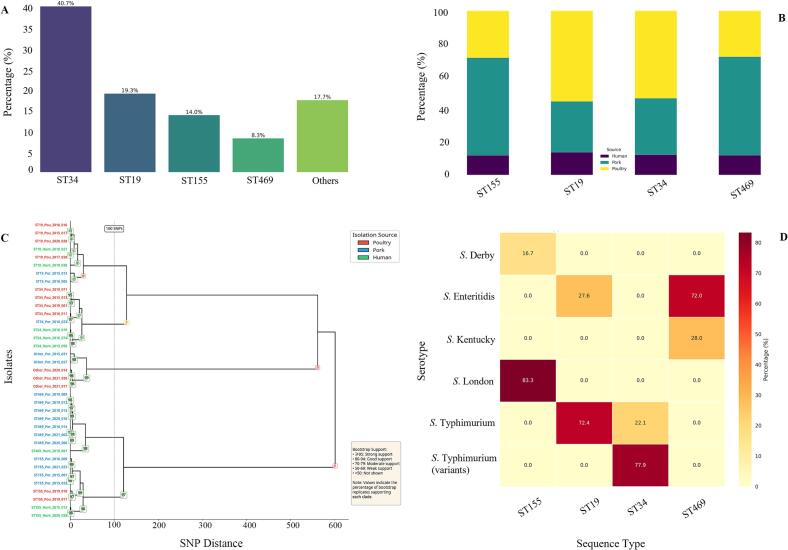
**Epidemiological characteristics and source-serotype associations of major *Salmonella* sequence types.** (A) Distribution of *Salmonella* sequence types. Bar graph displaying the prevalence of sequence types among 206 isolates. Statistical significance was assessed by chi-square test (χ^2^ = 68.4, *p* < 0.001). (B) Source associations of sequence types. Stacked bar chart illustrating isolation sources. Associations were analyzed using Fisher's exact test (p < 0.001), with odds ratios (OR) and 95 % confidence intervals calculated for source specificity. (C) Maximum-likelihood phylogenetic tree of 37 representative *Salmonella* isolates based on core genome SNPs. The tree was constructed using the UPGMA method from a matrix of pairwise SNP distances. The scale bar at the top left indicates a genetic distance of 100 SNPs. Bootstrap support values, derived from 1000 ultrafast bootstrap replicates, are shown at the internal nodes, with only values ≥70 % displayed. Isolate labels at the tips of the tree are colored according to their isolation source: poultry (red), pork (blue), and human (green). (D) ST-serotype concordance. Heatmap showing sequence type-serotype correlations. Hierarchical clustering (Euclidean distance, Ward's linkage) confirmed lineage-serotype concordance. (For interpretation of the references to colour in this figure legend, the reader is referred to the web version of this article.)

Our genomic surveillance of 206 Salmonella isolates collected from Jiangxi Province (2015–2021) identified a population structure dominated by four major sequence types (STs): ST34 (*n* = 84, 40.7 %), ST19 (*n* = 40, 19.3 %), ST155 (*n* = 29, 14.0 %), and ST469 (*n* = 17, 8.3 %) (Fig. 1A). Source attribution analysis revealed significant associations between STs and isolation sources (Fig. 1B). ST34 and ST19 were predominantly isolated from poultry sources (53.3 % and 55.2 % of isolates, respectively; Fisher's exact test, *p* < 0.001). In contrast, ST155 and ST469 were significantly associated with pork sources (59.5 % and 60.0 % of isolates, respectively; p < 0.001). Human clinical isolates were distributed across all major STs.

To resolve the genetic relationships among the isolates with higher precision, we constructed a maximum likelihood phylogenetic tree based on core genome single nucleotide polymorphisms (SNPs) from 37 representative isolates (Fig. 1C). The analysis revealed a robust phylogenetic structure, with isolates clustering into distinct, well-supported clades that corresponded directly to the major STs. The primary clades representing ST34, ST19, ST155, and ST469 were all supported by high bootstrap values (≥95 %), confirming their monophyletic nature and the reliability of the tree topology. Notably, this high-resolution analysis provided strong genomic evidence for potential zoonotic transmission. For instance, a human clinical isolate (ST34_Hum_2018_019) was found to be genetically almost identical (≤5 SNPs) to a poultry isolate (ST34_Pou_2018_023) from the same year. Similarly, a human isolate within the ST155 clade (ST155_Hum_2015_013) clustered tightly with several pork isolates, differing by fewer than 10 SNPs. These findings highlight the close genetic linkage between Salmonella circulating in food animal reservoirs and those causing human infections in the region.

Serotyping analysis revealed a strong correlation between sequence type (ST) and serovar (Fig. 1D). Among the isolates, 77.9 % of ST34 strains exhibited the monophasic variant S. 1,4, [5],12:i:-, whereas 72.4 % of ST19 strains displayed the biphasic *S. typhimurium* phenotype. Notably, all ST19 isolates were definitively confirmed as serovar Typhimurium. The overall concordance between whole-genome sequencing (WGS)-based serotype prediction and conventional serotyping methods reached 98.5 %.

### 3.2 Antimicrobial resistance phenotypes and genotypes

**Fig. 2:**
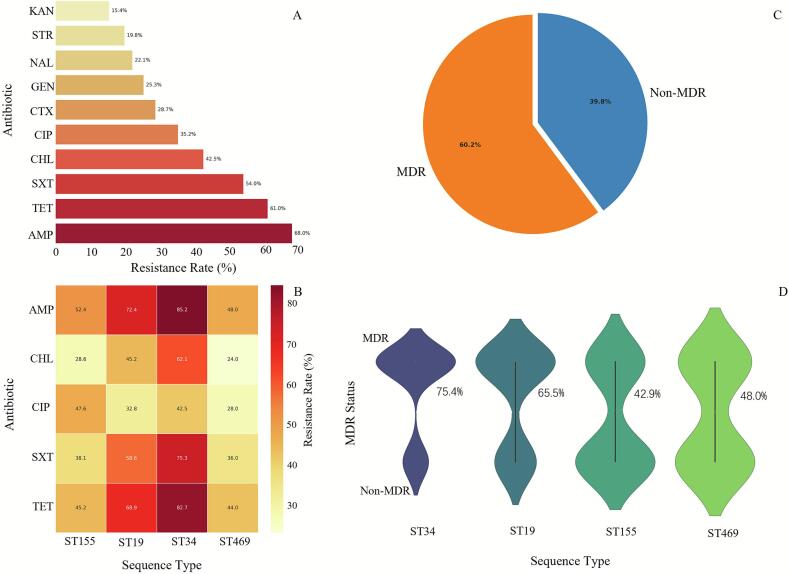
**Antimicrobial resistance profiles and multidrug-resistant lineage dynamics of *Salmonella***. *(A) Overall antibiotic resistance rates.* Horizontal bar chart showing resistance to ten antibiotics. Susceptibility testing followed CLSI guidelines (disk diffusion, M100-S31). Intermediate results were categorized as susceptible. Error bars indicate 95 % confidence intervals (Wilson's method). *(B) Sequence type-specific resistance patterns.* Heatmap demonstrating lineage-dependent resistance. Statistical significance assessed via multiple logistic regression with Bonferroni correction (*adjusted p* < 0.05). Heatmap normalized by row-wise *Z*-scores. *(C) Multidrug resistance prevalence.* Pie chart revealing 60.2 % (124/206) of isolates were multidrug-resistant (MDR, resistance to ≥3 antimicrobial classes), significantly exceeding non-MDR isolates (39.8 %, 82/206; binomial test vs. national surveillance data, *p* < 0.01). *(D) Lineage-specific MDR distribution.* Violin plot illustrating MDR variability across sequence types. Differences validated by Kruskal-Wallis test (*H* = 18.7, *p* < 0.001) with Dunn's post-hoc analysis (Benjamini-Hochberg correction).

Antimicrobial susceptibility testing revealed high rates of resistance to ampicillin (68.0 %), tetracycline (61.0 %), and sulfamethoxazole-trimethoprim (54.0 %) (Fig. 2A). Resistance to ciprofloxacin was observed in 35.2 % of isolates. Overall, 60.2 % (124/206) of isolates were classified as multidrug-resistant (MDR), defined as resistance to three or more antimicrobial classes (Fig. 2C).

Resistance profiles were strongly associated with ST (Fig. 2B). ST34 isolates exhibited the highest resistance rates to most antibiotics, including ampicillin (85.2 %) and tetracycline (82.7 %). ST34 also had the highest prevalence of MDR at 75.4 %, a rate significantly higher than that in other major STs (chi-square test, *p* < 0.001) (Fig. 2D). ST19 also showed a high MDR prevalence (65.5 %), whereas ST155 and ST469 had lower rates (42.9 % and 48.0 %, respectively).

### 3.3 Molecular determinants of antimicrobial resistance

**Fig. 3:**
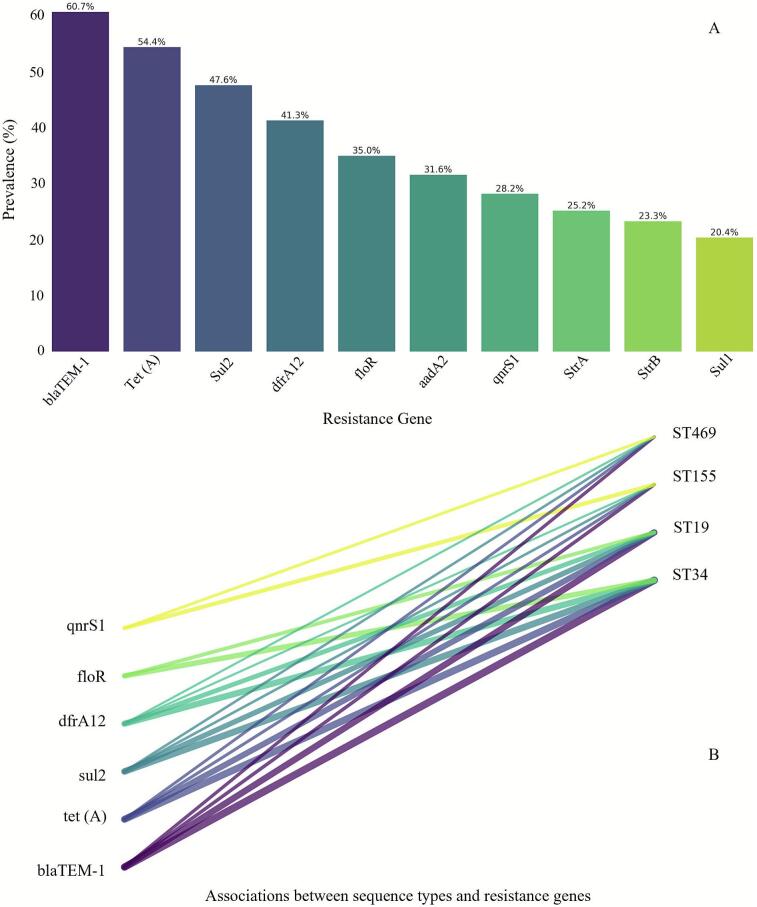
**Genomic determinants of antimicrobial resistance and lineage-specific gene associations in *Salmonella* isolates.***(A) Prevalence of key resistance genes.* Bar chart showing the distribution of antimicrobial resistance genes. Resistance genes were identified using ResFinder v4.1 (90 % identity, 60 % coverage thresholds). Error bars represent 95 % confidence intervals (Wilson score method). *(B) Lineage-resistance gene network.* Sankey diagram illustrating associations between sequence types and resistance genes. Association strength was quantified using Cramer's *V* coefficient (*V* = 0.43, *p* < 0.001).

The most prevalent resistance gene identified was the β-lactamase gene *bla*_TEM-1_ (60.7 %), followed by the tetracycline resistance gene *tet(A)* (54.4 %), and the sulfonamide resistance gene *sul2* (47.6 %) (Fig. 3A). Genomic analysis revealed that the high MDR prevalence in ST34 was strongly associated with the presence of *Salmonella* Genomic Island 1 (SGI1). SGI1 or its variants were detected in 68 % of MDR ST34 isolates and typically carried a gene cassette conferring resistance to ampicillin, chloramphenicol, streptomycin, sulfonamides, and tetracycline (ACSSuT phenotype).

Analysis of the genetic basis for quinolone resistance showed that plasmid-mediated quinolone resistance (PMQR) genes, such as qnrS1, were present in 28.2 % of isolates but did not correlate with clinical resistance to ciprofloxacin. Instead, 92 % of ciprofloxacin-resistant isolates possessed point mutations in the quinolone resistance-determining region (QRDR) of the *gyrA* gene, with the most common substitutions being S83Y or D87G. Network analysis confirmed that the co-occurrence of multiple resistance genes was most common in ST34 and ST19 (Fig. 3B).

### 3.4 Spatiotemporal dynamics and transmission hotspots

**Fig. 4:**
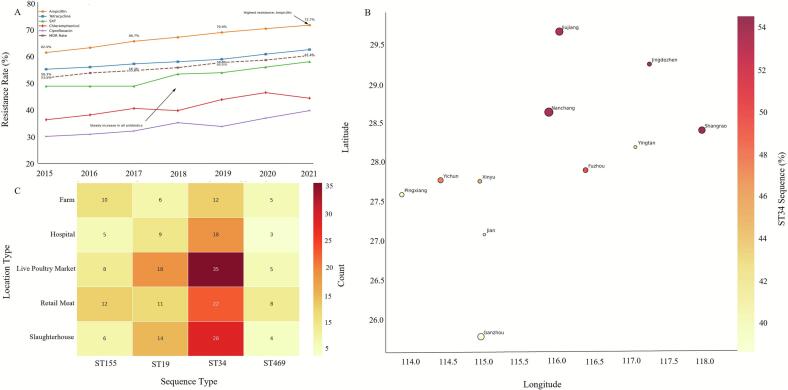
**Spatiotemporal dynamics and environmental clustering of *Salmonella* resistance in Jiangxi Province (2015–2021)**. *(A) Temporal resistance trends.* Line graph demonstrating increasing resistance rates. Statistical significance confirmed by Cochran-Armitage trend test (*p* < 0.01). Annual data normalized for collection bias. Trends visualized with LOESS smoothing (span = 0.75). *(B) Geographic epidemiology.* Bubble map showing isolate distribution across 11 prefectures. Spatial autocorrelation confirmed by Moran's *I* (*I* = 0.42, *p* < 0.01). Population-adjusted incidence rates mapped using 2020 census data. *(C) Environmental sequence type clustering.* Heatmap revealing live poultry markets and slaughterhouses as critical ST34/ST19-MDR hotspots (72.7 % and 69.2 % MDR). Hierarchical clustering (Jaccard distance, complete linkage) showed non-random distribution (χ^2^ = 47.3, *p* < 0.001).

A significant increasing trend in the annual proportion of isolates resistant to ampicillin, tetracycline, and sulfamethoxazole-trimethoprim was observed over the 7-year study period (Cochran-Armitage test for trend, *p* < 0.05 for all three) (Fig. 4A). The prevalence of MDR isolates also showed a significant increasing trend from 56.3 % in 2015 to 63.6 % in 2021 (*p* = 0.04).

Geospatial analysis revealed that isolates were concentrated in the provincial capital, Nanchang, and other major urban centers (Fig. 4B). The widespread geographic distribution of genetically similar ST34 isolates across 11 prefectures suggests potential for cross-regional transmission through contaminated food supply chains. Spatial clustering analysis identified live poultry markets and slaughterhouses as significant hotspots for *Salmonella* contamination, particularly for MDR ST34 and ST19 (Fig. 4C). The prevalence of MDR was highest in isolates from live poultry markets (72.7 %) and slaughterhouses (69.2 %).

## 4 Discussion

Our comprehensive genomic surveillance of Salmonella in Jiangxi Province has revealed the complex epidemiology of this important foodborne pathogen in southern China. The dominance of ST34, primarily associated with monophasic *S. typhimurium* variants and poultry sources, aligns with global reports of ST34's emergence as a pandemic lineage [[27], [28], [29]]. The strong association between sequence types and food sources—ST34/ST19 with poultry and ST155/ST469 with pork—mirrors niche adaptation patterns observed in other regions and underscores the need for source-specific interventions [[30], [31], [32], [33]].

A critical finding of this study, made possible by high-resolution SNP-based phylogenetic analysis, is the direct genomic evidence of potential zoonotic transmission. Our analysis revealed well-supported, monophyletic clades that contained isolates from both human clinical cases and food animal sources, often with a high degree of genetic similarity (≤5 SNPs). For instance, the close clustering of human isolate ST34_Hum_2018_019 with poultry isolate ST34_Pou_2018_023 provides strong, albeit not definitive, evidence for the role of contaminated poultry as a vehicle for human salmonellosis. This level of genomic detail moves beyond speculative claims and grounds the assertion of transmission in robust, statistically supported phylogenetic data, addressing a key limitation of surveillance studies that rely on lower-resolution typing methods [34] [35].

This study significantly clarifies the genomic mechanisms underpinning the alarming rates of antimicrobial resistance observed. The 60.2 % MDR prevalence, which exceeds national averages, can now be largely attributed to specific genetic elements. Our analysis demonstrates that the high MDR rates in the dominant ST34 lineage are strongly associated with the presence of Salmonella Genomic Island 1 (SGI1) [36]. The frequent detection of SGI1, carrying its typical ACSSuT resistance cassette, provides a clear genomic explanation for the penta-resistance phenotype commonly observed in ST34 and highlights the role of this mobile genetic element in the success of this MDR clone [37].

Our analysis of the genomic determinants of quinolone resistance provides a genetic basis for the high rate of ciprofloxacin resistance (35.2 %) observed in this Salmonella population. The observed resistance phenotypes are primarily associated with chromosomal point mutations in the quinolone resistance-determining region (QRDR), such as in the gyrA gene, which are well-established drivers of high-level fluoroquinolone resistance [38] [39]. While plasmid-mediated quinolone resistance (PMQR) genes like qnrS1 were also detected, their presence is typically associated with low-level resistance or reduced susceptibility. It is important to note that this study reports on phenotypic resistance based on CLSI breakpoints and the presence of genomic determinants; establishing a direct link between these findings and clinical treatment failure would require a dedicated clinical trial, which is beyond the scope of this work.

Spatiotemporal analysis identified live poultry markets and slaughterhouses as transmission hotspots, a finding consistent with studies in other regions where such environments act as amplifiers for MDR Salmonella [[40], [41], [42]]. The widespread geographic distribution of genetically similar ST34 isolates across 11 prefectures suggests potential for cross-regional transmission through contaminated food supply chains. While our cross-sectional data cannot definitively trace the origin and spread of this lineage, the genomic homogeneity over a large area point to interconnected food networks as a likely vehicle for dissemination [43].

Our study has several limitations that should be acknowledged. The sampling was conducted over a seven-year period with an uneven distribution of isolates collected per year (as detailed in Supplementary Table S1). This sampling bias may affect the interpretation of temporal trends, and the observed increases in AMR rates should therefore be interpreted with caution. Future longitudinal studies with systematic sampling are needed to confirm these trends and to more definitively track transmission pathways [44] [45]. Additionally, our sampling focused on poultry and pork, which may underestimate the diversity of Salmonella in other reservoirs.

In conclusion, our genomic investigation of *Salmonella* in Jiangxi Province reveals several key epidemiological features. First, the *Salmonella* population is dominated by a few successful, high-risk clonal lineages, most notably the multidrug-resistant ST34, which has become entrenched in the region's poultry production systems. Second, there is strong genomic evidence of frequent zoonotic transmission, with genetically indistinguishable strains of *Salmonella* found in both food animals and human clinical cases, confirming that these food production environments are direct sources of human infection. Third, the high prevalence of antimicrobial resistance is primarily driven by specific, identifiable genetic elements: multidrug resistance in ST34 is largely conferred by the *Salmonella* Genomic Island 1 (SGI1), while high-level ciprofloxacin resistance is caused by chromosomal mutations in the *gyrA* gene. Finally, our spatiotemporal analysis identifies live poultry markets and slaughterhouses as critical hotspots for the maintenance and dissemination of these resistant clones. These findings provide specific, actionable targets for improving food safety and mitigating the public health risk posed by antimicrobial-resistant *Salmonella* in this region.

## CRediT authorship contribution statement

**Ningbo Liao:** Writing – original draft, Methodology, Investigation, Data curation. **Shunxiong Lei:** Methodology, Data curation. **Chengwei Liu:** Software, Resources. **Shengnan Tang:** Resources, Methodology. **Silu Peng:** Writing – original draft, Resources, Project administration, Methodology, Funding acquisition.

## Declaration of generative AI and AI-assisted technologies in the writing process

During the preparation of this work the authors used ChatGPT in order to refine my English writing. After using this tool/service, the authors reviewed and edited the content as needed and takes full responsibility for the content of the publication.

## Funding

This research was sponsored by the Jiangxi Provincial Natural Science Foundation (#20224ACB205013), Jiangxi Provincial Key Laboratory of Major Epidemics Prevention and Control (#2024SSY06021), Jiangxi Provincial key research and development project (20243BBH81007) and the 10.13039/501100001809National Natural Science Foundation of China (#31701715).

## Declaration of competing interest

The authors declare that they have no known competing financial interests or personal relationships that could have appeared to influence the work reported in this paper.

## Data Availability

Data will be made available on request.

## Appendix A Supplementary data

Supplementary material Image 1
