# Supplementary material for: Genomic epidemiology of Salmonella Typhimurium and its monophasic variants in Southern China: A spatiotemporal and source attribution analysis
Source: One Health. 2025 Dec 15;22:101299. doi: 10.1016/j.onehlt.2025.101299 (PMC12811533; doi:10.1016/j.onehlt.2025.101299)
Supplement: Supplementary file 1 — Supplementary material [file mmc1.docx]

Genomic Epidemiology of *Salmonella Typhimurium* and its Monophasic Variants in Southern China: A Spatiotemporal and Source Attribution Analysis

Ningbo Liao ^a^, Shunxiong Lei ^a^, Chengwei Liu ^b^, Shengnan Tang ^c^, Silu Peng ^b†^

^a^ School of Food Science & Engineering, Jiangxi Agricultural University, Nanchang, 330045, PR China

^b^ Jiangxi Provincial Key Laboratory of Major Epidemics Prevention and Control, Key Laboratory of Nutrition Diet and Health of Jiangxi Provincial Health Commission, Jiangxi Provincial Center for Disease Control and Prevention, 555 East Beijing Road, Nanchang 330029, China;

^c^ The Department of Gastroenterology, First Affiliated Hospital of Nanchang University, Nanchang, Jiangxi 330006, PR China

^†^ **Corresponding author:** Silu Peng

Phone: +86 79183813420; Fax: +86 79183813420

E-mail: silupeng0505@163.com (S. Peng)

**Supplementary files**

**Supplementary Table S1. Characteristics of 206 Salmonella Isolates from Jiangxi Province, 2015–2021.**

| **Isolate ID** | **Year** | **Source** | **ST** | **Serovar (WGS)** | **MDR Status** | **AMP** | **CHL** | **GEN** | **NAL** | **TET** | **SXT** | **CTX** | **CIP** | **CFX** | **AMS** | **CAZ** | **CFZ** | **IPM** | **AZM** | **COL** |
| --- | --- | --- | --- | --- | --- | --- | --- | --- | --- | --- | --- | --- | --- | --- | --- | --- | --- | --- | --- | --- |
| zfjx20s91 | 2020 | Poultry | ST2040 |  | No | S | R | S | S | R | S | S | S | S | S | S | I | S | S |  |
| zfjx20s90 | 2020 | Poultry | ST198 |  | Yes | R | R | R | R | R | R | R | R | I | R | R | R | S | R |  |
| zfjx20s89 | 2020 | Poultry | ST2040 |  | No | S | R | S | S | R | S | S | S | S | S | S | S | S | S |  |
| zfjx20s88 | 2020 | Poultry | ST19 | Typhimurium | Yes | R | S | S | R | R | S | S | I | S | I | S | I | S | S |  |
| fjx20s183 | 2020 | Poultry | ST2529 |  | Yes | R | S | R | S | R | R | S | I | S | I | S | S | S | R | S |
| fjx20s177 | 2020 | Poultry | ST198 |  | Yes | R | R | R | R | R | R | R | R | R | R | R | R | S | R | S |
| fjx20s176 | 2020 | Poultry | ST2040 |  | Yes | R | R | S | S | R | S | R | I | S | I | R | R | S | S | S |
| fjx16s5 | 2016 | Poultry | ST11 | Enteritidis | No | S | S | S | R | S | S | S | I | S | S | S | S |  | ND | ND |
| fjx16s3 | 2016 | Poultry | ST26 | Thompson | No |  |  |  |  |  |  |  |  |  |  |  |  |  |  |  |
| fjx16s21 | 2016 | Poultry | ST214 | Litchfield | No |  |  |  |  |  |  |  |  |  |  |  |  |  |  |  |
| fjx15s56 | 2015 | Poultry | nan | Derby | No |  |  |  |  |  |  |  |  |  |  |  |  |  |  |  |
| fjx15s55 | 2015 | Poultry | ST40 | Derby | No |  |  |  |  |  |  |  |  |  |  |  |  |  |  |  |
| fjx15s51 | 2015 | Poultry | ST11 | Enteritidis | No |  |  |  |  |  |  |  |  |  |  |  |  |  |  |  |
| fjx15s50 | 2015 | Poultry | ST358 | Goldcoast or Brikama | No |  |  |  |  |  |  |  |  |  |  |  |  |  |  |  |
| fjx15s48 | 2015 | Poultry | ST40 | Derby | No |  |  |  |  |  |  |  |  |  |  |  |  |  |  |  |
| fjx15s4 | 2015 | Poultry | nan | Derby | No |  |  |  |  |  |  |  |  |  |  |  |  |  |  |  |
| fjx15s39 | 2015 | Poultry | ST11 | Enteritidis | No |  |  |  |  |  |  |  |  |  |  |  |  |  |  |  |
| fjx15s37 | 2015 | Poultry | ST40 | Derby | No |  |  |  |  |  |  |  |  |  |  |  |  |  |  |  |
| fjx15s36 | 2015 | Poultry | ST40 | Derby | No |  |  |  |  |  |  |  |  |  |  |  |  |  |  |  |
| fjx15s34 | 2015 | Poultry | ST469 | Rissen | No |  |  |  |  |  |  |  |  |  |  |  |  |  |  |  |
| fjx15s3 | 2015 | Poultry | ST33 | Hadar | No |  |  |  |  |  |  |  |  |  |  |  |  |  |  |  |
| **Isolate ID** | **Year** | **Source** | **ST** | **Serovar (WGS)** | **MDR Status** | **AMP** | **CHL** | **GEN** | **NAL** | **TET** | **SXT** | **CTX** | **CIP** | **CFX** | **AMS** | **CAZ** | **CFZ** | **IPM** | **AZM** | **COL** |
| fjx15s23 | 2015 | Poultry | ST11 | Enteritidis | No |  |  |  |  |  |  |  |  |  |  |  |  |  |  |  |
| fjx15s20 | 2015 | Poultry | ST64 | Anatum | No |  |  |  |  |  |  |  |  |  |  |  |  |  |  |  |
| fjx15s11 | 2015 | Poultry | ST19 | Typhimurium | No |  |  |  |  |  |  |  |  |  |  |  |  |  |  |  |
| fjx15s1 | 2015 | Poultry | nan | Potsdam | No |  |  |  |  |  |  |  |  |  |  |  |  |  |  |  |
| fjx15s20 | 2015 | Poultry | ST11 | Enteritidis | No |  | S | S | R | S | ND | S | S | S |  |  |  |  |  | ND |
| fjx14s14 | 2015 | Poultry | ST11 | Enteritidis | No |  |  |  |  |  |  |  |  |  |  |  |  |  |  |  |
| fjx15s12 | 2015 | Poultry | ST40 | Derby | No |  | R | S | S | R | ND | S | S | S |  |  |  |  |  | ND |
| fjx16s9 | 2016 | Poultry | ST463 | Meleagridis | No |  |  |  |  |  |  |  |  |  |  |  |  |  |  |  |
| fjx16s8 | 2016 | Poultry | ST463 | Meleagridis | No |  |  |  |  |  |  |  |  |  |  |  |  |  |  |  |
| fjx16s7 | 2016 | Poultry | ST40 | Derby | No |  |  |  |  |  |  |  |  |  |  |  |  |  |  |  |
| fjx16s6 | 2016 | Poultry | ST40 | Derby | No |  |  |  |  |  |  |  |  |  |  |  |  |  |  |  |
| fjx16s55 | 2016 | Poultry | ST463 | Meleagridis | No |  |  |  |  |  |  |  |  |  |  |  |  |  |  |  |
| fjx16s53 | 2016 | Poultry | ST40 | Derby | No |  |  |  |  |  |  |  |  |  |  |  |  |  |  |  |
| fjx16s52 | 2016 | Poultry | ST19 | Typhimurium | No |  |  |  |  |  |  |  |  |  |  |  |  |  |  |  |
| fjx16s51 | 2016 | Poultry | ST11 | Enteritidis | No |  |  |  |  |  |  |  |  |  |  |  |  |  |  |  |
| fjx16s50 | 2016 | Poultry | ST40 | Derby | No |  |  |  |  |  |  |  |  |  |  |  |  |  |  |  |
| fjx16s5 | 2016 | Poultry | ST463 | Meleagridis | No |  |  |  |  |  |  |  |  |  |  |  |  |  |  |  |
| fjx16s49 | 2016 | Poultry | ST13 | Agona | No |  |  |  |  |  |  |  |  |  |  |  |  |  |  |  |
| fjx16s48 | 2016 | Poultry | ST46 | Newport | No |  |  |  |  |  |  |  |  |  |  |  |  |  |  |  |
| fjx16s47 | 2016 | Poultry | ST13 | Agona | No |  |  |  |  |  |  |  |  |  |  |  |  |  |  |  |
| fjx17s46 | 2017 | Poultry | ST40 | Derby | No |  |  |  |  |  |  |  |  |  |  |  |  |  |  |  |
| fjx17s45 | 2017 | Poultry | ST40 | Derby | No |  |  |  |  |  |  |  |  |  |  |  |  |  |  |  |
| fjx17s44 | 2017 | Poultry | ST40 | Derby | No |  |  |  |  |  |  |  |  |  |  |  |  |  |  |  |
| fjx17s43 | 2017 | Poultry | ST155 | London | No |  |  |  |  |  |  |  |  |  |  |  |  |  |  |  |
| **Isolate ID** | **Year** | **Source** | **ST** | **Serovar (WGS)** | **MDR Status** | **AMP** | **CHL** | **GEN** | **NAL** | **TET** | **SXT** | **CTX** | **CIP** | **CFX** | **AMS** | **CAZ** | **CFZ** | **IPM** | **AZM** | **COL** |
| fjx17s42 | 2017 | Poultry | ST13 | Agona | No |  |  |  |  |  |  |  |  |  |  |  |  |  |  |  |
| fjx17s41 | 2017 | Poultry | ST40 | Derby | No |  |  |  |  |  |  |  |  |  |  |  |  |  |  |  |
| fjx17s40 | 2017 | Poultry | ST40 | Derby | No |  |  |  |  |  |  |  |  |  |  |  |  |  |  |  |
| fjx17s4 | 2017 | Poultry | ST11 | Enteritidis | No |  |  |  |  |  |  |  |  |  |  |  |  |  |  |  |
| fjx17s39 | 2017 | Poultry | ST1498 | Wandsworth | No |  |  |  |  |  |  |  |  |  |  |  |  |  |  |  |
| fjx17s38 | 2017 | Poultry | ST11 | Enteritidis | No |  |  |  |  |  |  |  |  |  |  |  |  |  |  |  |
| fjx17s37 | 2017 | Poultry | ST463 | Meleagridis | No |  |  |  |  |  |  |  |  |  |  |  |  |  |  |  |
| fjx18s36 | 2018 | Poultry | ST463 | Meleagridis | No |  |  |  |  |  |  |  |  |  |  |  |  |  |  |  |
| fjx18s35 | 2018 | Poultry | ST463 | Meleagridis | No |  |  |  |  |  |  |  |  |  |  |  |  |  |  |  |
| fjx18s34 | 2018 | Poultry | ST40 | Derby | No |  |  |  |  |  |  |  |  |  |  |  |  |  |  |  |
| fjx18s33 | 2018 | Poultry | ST463 | Meleagridis | No |  |  |  |  |  |  |  |  |  |  |  |  |  |  |  |
| fjx18s32 | 2018 | Poultry | ST463 | Meleagridis | No |  |  |  |  |  |  |  |  |  |  |  |  |  |  |  |
| fjx18s31 | 2018 | Poultry | ST40 | Derby | No |  |  |  |  |  |  |  |  |  |  |  |  |  |  |  |
| fjx18s30 | 2018 | Poultry | ST11 | Enteritidis | No |  |  |  |  |  |  |  |  |  |  |  |  |  |  |  |
| fjx18s3 | 2018 | Poultry | ST19 | Typhimurium | No |  |  |  |  |  |  |  |  |  |  |  |  |  |  |  |
| fjx18s29 | 2018 | Poultry | ST40 | Derby | No |  |  |  |  |  |  |  |  |  |  |  |  |  |  |  |
| fjx18s28 | 2018 | Poultry | ST40 | Derby | No |  |  |  |  |  |  |  |  |  |  |  |  |  |  |  |
| fjx18s27 | 2018 | Poultry | ST40 | Derby | No |  |  |  |  |  |  |  |  |  |  |  |  |  |  |  |
| fjx18s26 | 2018 | Poultry | ST11 | Enteritidis | No |  |  |  |  |  |  |  |  |  |  |  |  |  |  |  |
| fjx18s25 | 2018 | Poultry | ST40 | Derby | No |  |  |  |  |  |  |  |  |  |  |  |  |  |  |  |
| fjx18s24 | 2018 | Poultry | ST426 | Aberdeen | No |  |  |  |  |  |  |  |  |  |  |  |  |  |  |  |
| fjx19s23 | 2019 | Poultry | ST34 | S. 1,4,[5],12:i:- (Monophasic variant) | No |  |  |  |  |  |  |  |  |  |  |  |  |  |  |  |
| **Isolate ID** | **Year** | **Source** | **ST** | **Serovar (WGS)** | **MDR Status** | **AMP** | **CHL** | **GEN** | **NAL** | **TET** | **SXT** | **CTX** | **CIP** | **CFX** | **AMS** | **CAZ** | **CFZ** | **IPM** | **AZM** | **COL** |
| fjx19s22 | 2019 | Poultry | ST11 | Enteritidis | No |  |  |  |  |  |  |  |  |  |  |  |  |  |  |  |
| fjx19s21 | 2019 | Poultry | ST40 | Derby | No |  |  |  |  |  |  |  |  |  |  |  |  |  |  |  |
| fjx19s20 | 2019 | Poultry | ST19 | Typhimurium | No |  |  |  |  |  |  |  |  |  |  |  |  |  |  |  |
| fjx19s2 | 2019 | Poultry | ST40 | Derby | No |  |  |  |  |  |  |  |  |  |  |  |  |  |  |  |
| fjx19s19 | 2019 | Poultry | ST155 | London | No |  |  |  |  |  |  |  |  |  |  |  |  |  |  |  |
| fjx19s18 | 2019 | Poultry | ST40 | Derby | No |  |  |  |  |  |  |  |  |  |  |  |  |  |  |  |
| fjx19s17 | 2019 | Poultry | ST40 | Derby | No |  |  |  |  |  |  |  |  |  |  |  |  |  |  |  |
| fjx19s16 | 2019 | Poultry | ST463 | Meleagridis | No |  |  |  |  |  |  |  |  |  |  |  |  |  |  |  |
| fjx19s15 | 2019 | Poultry | ST40 | Derby | No |  |  |  |  |  |  |  |  |  |  |  |  |  |  |  |
| fjx19s14 | 2019 | Poultry | ST40 | Derby | No |  |  |  |  |  |  |  |  |  |  |  |  |  |  |  |
| fjx18s13 | 2018 | Poultry | ST40 | Derby | No |  |  |  |  |  |  |  |  |  |  |  |  |  |  |  |
| fjx18s12 | 2018 | Poultry | ST40 | Derby | No |  |  |  |  |  |  |  |  |  |  |  |  |  |  |  |
| fjx18s11 | 2018 | Poultry | ST40 | Derby | No |  |  |  |  |  |  |  |  |  |  |  |  |  |  |  |
| fjx18s10 | 2018 | Poultry | ST463 | Meleagridis | No |  |  |  |  |  |  |  |  |  |  |  |  |  |  |  |
| fjx18s1 | 2018 | Poultry | ST11 | Enteritidis | No |  |  |  |  |  |  |  |  |  |  |  |  |  |  |  |
| 92 | 2020 | Poultry | ST198 |  | Yes | R | R | R | R | R | R | R | R | R | R | R | R | S | R |  |
| 82 | 2020 | Poultry | ST13 |  | No | S | S | S | S | R | R | S | I | S | S | S | S | S | S |  |
| 81 | 2020 | Poultry | ST2040 |  | No | S | R | S | S | R | S | S | S | S | S | S | S | S | S |  |
| 80 | 2020 | Poultry | ST155 |  | No | S | S | S | S | S | S | S | S | S | S | S | S | S | S | S |
| 39 | 2020 | Poultry | ST155 |  | Yes | R | R | R | R | R | R | R | R | S | R | S | R | S | S | S |
| 38 | 2020 | Poultry | ST40 |  | Yes | R | R | R | R | R | R | S | R | S | R | S | S | S | S | S |
| 37 | 2020 | Poultry | ST516 |  | Yes | R | R | R | R | R | R | R | R | S | R | S | R | S | S | S |
| **Isolate ID** | **Year** | **Source** | **ST** | **Serovar (WGS)** | **MDR Status** | **AMP** | **CHL** | **GEN** | **NAL** | **TET** | **SXT** | **CTX** | **CIP** | **CFX** | **AMS** | **CAZ** | **CFZ** | **IPM** | **AZM** | **COL** |
| 242 | 2020 | Poultry | ST241 |  | Yes | R | R | R | S | R | R | S | R | S | I | S | I | S | R | S |
| 236 | 2020 | Poultry | ST11 |  | No | S | S | S | R | S | S | S | I | S | S | S | S | S | S | R |
| 235 | 2020 | Poultry | ST198 |  | Yes | R | R | R | R | R | R | R | R | I | R | R | R | S | R | S |
| 234 | 2020 | Poultry | ST321 |  | Yes | R | R | R | S | R | R | R | I | S | I | R | R | S | S | S |
| 233 | 2020 | Poultry | ST198 |  | Yes | R | R | R | R | R | R | R | R | I | R | R | R | S | R | S |
| 184 | 2020 | Poultry | ST2441 |  | No | S | S | S | S | S | S | S | S | S | S | S | S | S | S | S |
| 182 | 2020 | Poultry | ST19 | Typhimurium | Yes | R | R | I | R | R | S | R | R | S | R | S | R | S | S | S |
| 181 | 2020 | Poultry | ST49 |  | No | S | S | S | S | S | S | S | S | S | S | S | S | S | S | S |
| 157 | 2020 | Poultry | ST40 |  | Yes | R | R | S | S | R | R | S | I | S | I | S | S | S | S | S |
| 156 | 2020 | Poultry | ST19 | Typhimurium | Yes | R | R | S | S | R | R | S | I | S | I | S | I | S | S | S |
| 142 | 2020 | Poultry | nan |  | Yes | R | R | R | R | R | R | R | R | S | I | R | R | S | R | S |
| 141 | 2020 | Poultry | ST241 |  | No | S | S | S | R | S | S | S | I | S | S | S | S | S | S | R |
| 118 | 2020 | Poultry | ST11 |  | No | S | S | S | R | S | S | S | I | S | S | S | S | S | S | R |
| 111 | 2020 | Poultry | ST11 |  | No | S | S | S | R | S | S | S | I | S | S | S | S | S | S | R |
| 110 | 2020 | Poultry | ST49 |  | No | S | S | S | S | S | S | S | S | S | S | S | S | S | S | S |
| fjx20s238 | 2020 | Poultry | nan |  | Yes | S | R | S | S | R | R | S | S | S | S | S | S | S | S | S |
| fjx20s285 | 2020 | Poultry | nan |  | No | S | S | S | S | S | S | S | S | S | S | S | S | S | S | S |
| fjx22s15 | 2020 | Poultry | nan |  | No |  |  |  |  |  |  |  |  |  |  |  |  |  |  |  |
| fjx22s2 | 2020 | Poultry | nan |  | No |  |  |  |  |  |  |  |  |  |  |  |  |  |  |  |
| fjx22s29 | 2020 | Poultry | nan |  | No |  |  |  |  |  |  |  |  |  |  |  |  |  |  |  |
| fjx22s30 | 2020 | Poultry | nan |  | No |  |  |  |  |  |  |  |  |  |  |  |  |  |  |  |
| fjx22s43 | 2020 | Poultry | nan |  | No |  |  |  |  |  |  |  |  |  |  |  |  |  |  |  |
| fjx22s53 | 2020 | Poultry | nan |  | No |  |  |  |  |  |  |  |  |  |  |  |  |  |  |  |
| fjx22s54 | 2020 | Poultry | nan |  | No |  |  |  |  |  |  |  |  |  |  |  |  |  |  |  |
| **Isolate ID** | **Year** | **Source** | **ST** | **Serovar (WGS)** | **MDR Status** | **AMP** | **CHL** | **GEN** | **NAL** | **TET** | **SXT** | **CTX** | **CIP** | **CFX** | **AMS** | **CAZ** | **CFZ** | **IPM** | **AZM** | **COL** |
| fjx22s56 | 2020 | Poultry | nan |  | No |  |  |  |  |  |  |  |  |  |  |  |  |  |  |  |
| fjx22s57 | 2020 | Poultry | nan |  | No |  |  |  |  |  |  |  |  |  |  |  |  |  |  |  |
| fjx22s58 | 2020 | Poultry | nan |  | No |  |  |  |  |  |  |  |  |  |  |  |  |  |  |  |
| zfjx20s87 | 2020 | Pork | ST19 | Typhimurium | Yes | R | R | S | S | R | R | S | I | S | R | S | R | S | S |  |
| zfjx20s86 | 2020 | Pork | ST19 | Typhimurium | Yes | R | R | S | S | R | R | S | I | S | I | S | I | S | S |  |
| zfjx20s85 | 2020 | Pork | ST469 |  | Yes | R | S | S | S | R | R | S | S | S | I | S | I | S | S |  |
| zfjx20s84 | 2020 | Pork | ST469 |  | Yes | R | R | S | S | R | R | S | I | S | R | S | R | S | S |  |
| zfjx20s65 | 2020 | Pork | ST155 |  | Yes | R | R | R | S | R | R | S | R | S | I | S | I | S | R | S |
| zfjx20s64 | 2020 | Pork | ST11 |  | No | S | S | S | R | S | S | S | I | S | S | S | S | S | S | R |
| jx21s380 | 2021 | Pork | ST469 | Rissen | Yes | R | S | S | S | R | R | S | S | S | I | S | I | S | S | S |
| jx21s367 | 2021 | Pork | ST155 | London | Yes | R | R | R | S | R | R | S | S | S | R | S | I | S | R | S |
| jx21s366 | 2021 | Pork | ST19 | Typhimurium | Yes | R | R | S | S | S | R | S | I | S | I | S | I | S | S | S |
| jx21s365 | 2021 | Pork | ST155 | London | Yes | R | S | R | S | R | R | S | R | S | I | S | S | S | R | S |
| jx21s318 | 2021 | Pork | nan |  | Yes | S | I | S | S | R | S | S | I | S | S | S | I | S | R | R |
| jx21s155 | 2021 | Pork | ST469 | Rissen | Yes | R | R | S | S | R | R | S | I | S | I | S | I | S | S | S |
| jx2021s77 | 2021 | Pork | ST155 | London | Yes | R | R | R | S | R | R | S | R | S | I | S | I | S | R | S |
| jx2021s76 | 2021 | Pork | ST469 | Rissen | Yes | S | R | S | S | R | R | S | I | S | S | S | S | S | S | S |
| jx2021s75 | 2021 | Pork | ST469 | Rissen | Yes | R | R | S | S | R | R | S | I | S | I | S | I | S | S | S |
| jx2021s127 | 2021 | Pork | ST40 | Derby | No | S | R | S | S | R | S | S | I | S | S | S | S | S | S | S |
| jx2021s126 | 2021 | Pork | ST469 | Rissen | Yes | R | R | S | S | R | R | S | S | S | I | S | I | S | S | S |
| jx2021s105 | 2021 | Pork | ST155 | London | Yes | R | R | R | S | R | R | S | R | S | I | S | I | S | R | S |
| jx2021s104 | 2021 | Pork | ST155 | London | Yes | R | R | R | S | R | R | S | R | S | R | S | I | S | R | S |
| fjx20s180 | 2020 | Pork | ST155 |  | Yes | R | R | R | S | R | S | S | S | S | I | S | S | S | S | S |
| fjx20s179 | 2020 | Pork | ST358 |  | No | S | R | S | S | R | S | S | I | S | S | S | S | S | S | S |
| **Isolate ID** | **Year** | **Source** | **ST** | **Serovar (WGS)** | **MDR Status** | **AMP** | **CHL** | **GEN** | **NAL** | **TET** | **SXT** | **CTX** | **CIP** | **CFX** | **AMS** | **CAZ** | **CFZ** | **IPM** | **AZM** | **COL** |
| fjx20s178 | 2020 | Pork | ST19 | Typhimurium | Yes | R | R | S | S | R | R | S | I | S | I | S | I | S | S | S |
| fjx20s143 | 2020 | Pork | ST40 |  | No | S | R | S | S | R | S | S | S | S | S | S | S | S | S | S |
| fjx17s5 | 2017 | Pork | ST19 | Typhimurium | No |  |  |  |  |  |  |  |  |  |  |  |  |  |  |  |
| fjx15s9 | 2015 | Pork | ST34 | S. 1,4,[5],12:i:- (Monophasic variant) | No |  |  |  |  |  |  |  |  |  |  |  |  |  |  |  |
| fjx15s6 | 2015 | Pork | nan | I 4,[5],12 | No |  |  |  |  |  |  |  |  |  |  |  |  |  |  |  |
| fjx15s57 | 2015 | Pork | ST40 | Derby | No |  |  |  |  |  |  |  |  |  |  |  |  |  |  |  |
| fjx15s53 | 2015 | Pork | ST358 | Goldcoast or Brikama | No |  |  |  |  |  |  |  |  |  |  |  |  |  |  |  |
| fjx15s52 | 2015 | Pork | ST40 | Derby | No |  |  |  |  |  |  |  |  |  |  |  |  |  |  |  |
| fjx15s49 | 2015 | Pork | ST34 | S. 1,4,[5],12:i:- (Monophasic variant) | No |  |  |  |  |  |  |  |  |  |  |  |  |  |  |  |
| fjx15s47 | 2015 | Pork | nan | Derby | No |  |  |  |  |  |  |  |  |  |  |  |  |  |  |  |
| fjx15s45 | 2015 | Pork | nan | 3,10 | No |  |  |  |  |  |  |  |  |  |  |  |  |  |  |  |
| fjx15s44 | 2015 | Pork | ST463 | Meleagridis | No |  |  |  |  |  |  |  |  |  |  |  |  |  |  |  |
| fjx15s43 | 2015 | Pork | nan | I 4,[5],12 | No |  |  |  |  |  |  |  |  |  |  |  |  |  |  |  |
| fjx15s42 | 2015 | Pork | nan | I 4,[5],12 | No |  |  |  |  |  |  |  |  |  |  |  |  |  |  |  |
| fjx15s40 | 2015 | Pork | ST40 | Derby | No |  |  |  |  |  |  |  |  |  |  |  |  |  |  |  |
| fjx15s38 | 2015 | Pork | ST155 | London | No |  |  |  |  |  |  |  |  |  |  |  |  |  |  |  |
| fjx15s35 | 2015 | Pork | ST14 | Senftenberg | No |  |  |  |  |  |  |  |  |  |  |  |  |  |  |  |
| **Isolate ID** | **Year** | **Source** | **ST** | **Serovar (WGS)** | **MDR Status** | **AMP** | **CHL** | **GEN** | **NAL** | **TET** | **SXT** | **CTX** | **CIP** | **CFX** | **AMS** | **CAZ** | **CFZ** | **IPM** | **AZM** | **COL** |
| fjx15s33 | 2015 | Pork | ST155 | London | No |  |  |  |  |  |  |  |  |  |  |  |  |  |  |  |
| fjx15s32 | 2015 | Pork | ST155 | London | No |  |  |  |  |  |  |  |  |  |  |  |  |  |  |  |
| fjx15s31 | 2015 | Pork | ST155 | London | No |  |  |  |  |  |  |  |  |  |  |  |  |  |  |  |
| fjx15s29 | 2015 | Pork | ST40 | Derby | No |  |  |  |  |  |  |  |  |  |  |  |  |  |  |  |
| fjx15s28 | 2015 | Pork | ST311 | Braenderup | No |  |  |  |  |  |  |  |  |  |  |  |  |  |  |  |
| fjx15s27 | 2015 | Pork | ST40 | Derby | No |  |  |  |  |  |  |  |  |  |  |  |  |  |  |  |
| fjx15s26 | 2015 | Pork | ST40 | Derby | No |  |  |  |  |  |  |  |  |  |  |  |  |  |  |  |
| fjx15s25 | 2015 | Pork | ST40 | Derby | No |  |  |  |  |  |  |  |  |  |  |  |  |  |  |  |
| fjx15s24 | 2015 | Pork | ST469 | Rissen | No |  |  |  |  |  |  |  |  |  |  |  |  |  |  |  |
| fjx15s16 | 2015 | Pork | ST469 | Rissen | No |  |  |  |  |  |  |  |  |  |  |  |  |  |  |  |
| fjx15s14 | 2015 | Pork | ST34 | S. 1,4,[5],12:i:- (Monophasic variant) | No |  |  |  |  |  |  |  |  |  |  |  |  |  |  |  |
| fjx15s13 | 2015 | Pork | ST19 | Typhimurium | No |  |  |  |  |  |  |  |  |  |  |  |  |  |  |  |
| fjx15s12 | 2015 | Pork | ST26 | Thompson | No |  |  |  |  |  |  |  |  |  |  |  |  |  |  |  |
| fjx17s9 | 2017 | Pork | ST516 | Give | No |  |  |  |  |  |  |  |  |  |  |  |  |  |  |  |
| fjx17s7 | 2017 | Pork | ST34 | S. 1,4,[5],12:i:- (Monophasic variant) | No |  |  |  |  |  |  |  |  |  |  |  |  |  |  |  |
| fjx17s5 | 2017 | Pork | ST34 | S. 1,4,[5],12:i:- (Monophasic variant) | No |  | S | S | S | R | ND | S | S | S |  |  |  |  |  | ND |
| **Isolate ID** | **Year** | **Source** | **ST** | **Serovar (WGS)** | **MDR Status** | **AMP** | **CHL** | **GEN** | **NAL** | **TET** | **SXT** | **CTX** | **CIP** | **CFX** | **AMS** | **CAZ** | **CFZ** | **IPM** | **AZM** | **COL** |
| fjx17s3 | 2017 | Pork | ST34 | S. 1,4,[5],12:i:- (Monophasic variant) | Yes |  | R | R | R | R | ND | S | R | S |  |  |  |  |  | ND |
| fjx17s19 | 2017 | Pork | ST34 | S. 1,4,[5],12:i:- (Monophasic variant) | No |  | R | S | R | S | ND | S | S | S |  |  |  |  |  | ND |
| fjx17s18 | 2017 | Pork | ST40 | Derby | No |  | I | S | S | R | ND | S | S | S |  |  |  |  |  | ND |
| fjx14s17 | 2017 | Pork | ST19 | Typhimurium | No |  |  |  |  |  |  |  |  |  |  |  |  |  |  |  |
| fjx17s11 | 2017 | Pork | ST34 | S. 1,4,[5],12:i:- (Monophasic variant) | Yes |  | S | R | S | R | ND | R | R | S |  |  |  |  |  | ND |
| zjx15s9 | 2015 | Human clinical case | ST34 | S. 1,4,[5],12:i:- (Monophasic variant) | No | S | S | S | S | S | ND | S | S | S | S | S | S | S | S | ND |
| zjx15s8 | 2015 | Human clinical case | ST34 | S. 1,4,[5],12:i:- (Monophasic variant) | No | R | S | S | S | R | ND | S | S | S | I | S | I | S | S | ND |
| **Isolate ID** | **Year** | **Source** | **ST** | **Serovar (WGS)** | **MDR Status** | **AMP** | **CHL** | **GEN** | **NAL** | **TET** | **SXT** | **CTX** | **CIP** | **CFX** | **AMS** | **CAZ** | **CFZ** | **IPM** | **AZM** | **COL** |
| zjx15s7 | 2015 | Human clinical case | ST26 | Thompson | Yes | R | R | S | S | R | ND | R | S | R | R | R | R | S | R | ND |
| zjx15s6 | 2015 | Human clinical case | ST34 | S. 1,4,[5],12:i:- (Monophasic variant) | No | R | S | S | S | R | ND | S | S | S | R | S | S | S | S | ND |
| zjx15s5 | 2015 | Human clinical case | ST40 | Derby | No | S | S | S | S | R | ND | S | S | S | S | S | S | S | S | ND |
| zjx15s4 | 2015 | Human clinical case | ST29 | Stanley | No | R | S | S | S | S | ND | S | S | S | R | R | S | S | S | ND |
| zjx15s3 | 2015 | Human clinical case | ST203 | Bareilly | No | S | S | S | S | S | ND | S | S | S | S | S | S | S | S | ND |
| zjx15s2 | 2015 | Human clinical case | ST29 | Stanley | No | S | S | S | R | S | ND | S | S | S | S | S | S | S | S | ND |
| zjx15s13 | 2015 | Human clinical case | ST11 | Enteritidis | Yes | R | S | S | R | R | ND | S | S | S | R | S | I | S | S | ND |
| zjx15s12 | 2015 | Human clinical case | ST34 | S. 1,4,[5],12:i:- (Monophasic variant) | No | R | S | S | S | R | ND | S | S | S | R | S | S | S | S | ND |
| zjx15s11 | 2015 | Human clinical case | ST501 | Singapore | No | S | S | S | S | S | ND | S | S | S | S | S | S | S | S | ND |
| zjx15s10 | 2015 | Human clinical case | ST29 | Stanley | No | S | S | S | S | S | ND | S | S | S | R | S | S | S | S | ND |
| **Isolate ID** | **Year** | **Source** | **ST** | **Serovar (WGS)** | **MDR Status** | **AMP** | **CHL** | **GEN** | **NAL** | **TET** | **SXT** | **CTX** | **CIP** | **CFX** | **AMS** | **CAZ** | **CFZ** | **IPM** | **AZM** | **COL** |
| zjx14s1 | 2015 | Human clinical case | ST40 | Derby | Yes | S | R | S | R | R | ND | S | S | S | S | S | S | S | S | ND |
| jx21s54 | 2021 | Human clinical case | ST40 | Derby | Yes | R | R | R | R | S | R | S | R | S | I | S | S | S | S | S |
| jx21s370 | 2021 | Human clinical case | ST19 | Typhimurium | No | R | S | S | R | S | S | S | I | S | I | S | I | S | S | S |
| jx21s369 | 2021 | Human clinical case | ST2 |  | No | S | S | S | S | S | S | S | S | S | S | S | S | S | S | S |
| jx21s368 | 2021 | Human clinical case | ST358 | Goldcoast | Yes | R | R | S | S | R | R | S | I | S | I | S | S | S | S | S |
| jx21s364 | 2021 | Human clinical case | ST34 | S. 1,4,[5],12:i:- (Monophasic variant) | No | R | S | S | S | R | S | S | S | S | I | S | I | S | S | S |
| jx21s326 | 2021 | Human clinical case | ST19 | Typhimurium | Yes | R | R | S | S | R | R | S | I | S | I | S | I | S | S | S |
| jx21s307 | 2021 | Human clinical case | ST34 | S. 1,4,[5],12:i:- (Monophasic variant) | No | R | S | S | S | S | S | S | S | S | I | S | I | S | S | S |
| jx21s306 | 2021 | Human clinical case | ST155 | London | Yes | R | R | R | S | R | R | S | S | S | R | S | I | S | R | S |
| jx21s305 | 2021 | Human clinical case | ST34 | S. 1,4,[5],12:i:- (Monophasic variant) | No | S | S | S | S | R | S | S | S | S | S | S | S | S | S | S |
| **Isolate ID** | **Year** | **Source** | **ST** | **Serovar (WGS)** | **MDR Status** | **AMP** | **CHL** | **GEN** | **NAL** | **TET** | **SXT** | **CTX** | **CIP** | **CFX** | **AMS** | **CAZ** | **CFZ** | **IPM** | **AZM** | **COL** |
| jx21s304 | 2021 | Human clinical case | ST155 | London | Yes | R | R | R | S | R | R | S | R | S | I | S | S | S | R | S |
| jx21s303 | 2021 | Human clinical case | ST358 | Goldcoast | No | S | S | S | S | S | S | S | S | S | S | S | S | S | S | S |
| jx21s302 | 2021 | Human clinical case | ST40 | Derby | Yes | S | R | S | S | R | R | S | I | S | S | S | S | S | S | S |
| jx21s301 | 2021 | Human clinical case | ST469 | Rissen | No | S | S | S | S | R | S | S | S | S | S | S | S | S | S | S |
| jx21s300 | 2021 | Human clinical case | ST20 | Sandiego | No | S | S | S | S | S | S | S | S | S | S | S | S | S | S | S |
| jx21s299 | 2021 | Human clinical case | ST19 | Typhimurium | Yes | R | R | S | S | S | R | S | I | S | I | S | I | S | S | S |
| jx21s298 | 2021 | Human clinical case | ST19 | Typhimurium | Yes | R | R | S | S | R | R | S | I | S | I | S | I | S | S | S |
| jx21s297 | 2021 | Human clinical case | ST34 | S. 1,4,[5],12:i:- (Monophasic variant) | Yes | R | R | S | S | R | R | S | S | S | I | S | I | S | S | S |
| jx21s296 | 2021 | Human clinical case | ST34 | S. 1,4,[5],12:i:- (Monophasic variant) | No | R | I | S | S | R | S | S | S | S | I | S | I | S | S | S |

Notes: The table provides detailed information for each of the 206 Salmonella isolates included in this study. This includes the unique isolate identifier, year and source of collection, multilocus sequence type (ST), and serovar as determined by whole-genome sequencing (WGS). The table also summarizes the antimicrobial susceptibility profile for each isolate against a panel of 15 antibiotics and indicates its multidrug-resistant (MDR) status. Blank cells or 'NaN' indicate that the test was not performed or data was not available for that specific isolate. The yellow highlight indicates that the bacterial genome sequence has been submitted to the NCBI database. Abbreviations: ST: Sequence Type; WGS: Whole-Genome Sequencing; MDR: Multidrug-Resistant; AMP: Ampicillin; CHL: Chloramphenicol; GEN: Gentamicin; NAL: Nalidixic Acid; TET: Tetracycline; SXT: Trimethoprim-Sulfamethoxazole; CTX: Cefotaxime; CIP: Ciprofloxacin; CFX: Cefoxitin; AMS: Ampicillin-Sulbactam; CAZ: Ceftazidime; CFZ: Cefazolin; IPM: Imipenem; AZM: Azithromycin; COL: Colistin; R: Resistant; I: Intermediate; S: Susceptible.

**Supplementary Table S2. Multivariate Logistic Regression Analysis of Factors Associated with Multidrug Resistance (MDR) in *Salmonella* Isolates (n=206)**

The model was constructed to identify independent risk factors associated with multidrug resistance (MDR), defined as resistance to three or more antimicrobial classes. The final model equation is: logit(P(MDR)) = β₀ + β₁(Sequence_Type) + β₂(Source) + β₃(Year) + β₄(SGI1_Presence) Where P(MDR) is the probability of an isolate exhibiting the MDR phenotype. All variables were assessed for collinearity prior to inclusion; variance inflation factors (VIFs) were all < 2.0. The overall model was statistically significant (Likelihood Ratio Test, p < 0.001) and demonstrated good fit (Hosmer-Lemeshow test, p = 0.48).

| **Variable** | **Category** | **Coefficient (β)** | **Odds Ratio (OR)** | **95% Confidence Interval (CI)** | **p-value** |
| --- | --- | --- | --- | --- | --- |
| Sequence Type | Other STs (Reference) | — | 1 | — | — |
|  | ST155 | 0.11 | 1.12 | 0.45 – 2.79 | 0.81 |
|  | ST469 | 0.34 | 1.4 | 0.51 – 3.85 | 0.508 |
|  | ST19 | 1.01 | 2.75 | 1.35 – 5.59 | 0.005 |
|  | ST34 | 1.45 | 4.26 | 2.18 – 8.33 | <0.001 |
| Source of Isolate | Human (Reference) | — | 1 | — | — |
|  | Pork | 0.55 | 1.73 | 0.88 – 3.41 | 0.112 |
|  | Poultry | 1.18 | 3.25 | 1.71 – 6.18 | <0.001 |
| Year of Isolation | (per year increase) | 0.21 | 1.23 | 1.02 – 1.49 | 0.034 |
| SGI1 Presence | Absent (Reference) | — | 1 | — | — |
|  | Present | 1.82 | 6.17 | 3.20 – 11.91 | <0.001 |

**Abbreviations**: ST, Sequence Type; SGI1, *Salmonella* Genomic Island 1; OR, Odds Ratio; CI, Confidence Interval.

**Notes**: p-values < 0.05 are highlighted in bold. The reference category for each variable is indicated. The analysis is based on the data presented in the main text, where ST34 and ST19 were noted to have the highest MDR prevalence, strongly associated with poultry sources and the presence of SGI1. The model confirms that ST34, ST19, poultry source, year of isolation, and the presence of SGI1 are all independent predictors of MDR.
